# A32 CATHELICIDIN REGULATES GOBLET CELL MUCUS SECRETION DURING *CITROBACTER RODENTIUM*-INDUCED COLITIS

**DOI:** 10.1093/jcag/gwad061.032

**Published:** 2024-02-14

**Authors:** E R Cobo, G Blyth, F Fiorani, P Lahiri, A Herik, A Dufour, K Chadee

**Affiliations:** University of Calgary, Calgary, AB, Canada; University of Calgary, Calgary, AB, Canada; University of Calgary, Calgary, AB, Canada; University of Calgary, Calgary, AB, Canada; University of Calgary, Calgary, AB, Canada; University of Calgary, Calgary, AB, Canada; University of Calgary, Calgary, AB, Canada

## Abstract

**Background:**

Colonic goblet cells by secreting Muc2 mucin and specific proteins is critical for physically entrapping and expelling invading enteropathogens. Thus, is not surprising that *Muc2*^*-/-*^ littermates exhibit increased susceptibility to attaching/effacing *Citrobacter rodentium* colonization. The colonic epithelium also secretes small cathelicidin peptide, which potentially interacts intimately with goblet cells and was presumed to accumulate within the sterile inner mucus layer as a simple antimicrobial peptide defense.

**Aims:**

To determine the effects of cathelicidin on mucin secretion in goblet cells during *C. rodentium*-induced colitis and the impact on the mucus barrier defense.

**Methods:**

We used cathelicidin-deficient (*Camp*^*-/-*^) mice, mouse colonoids and human colonic LS174T like-goblet epithelial cells to elucidate the mechanisms by which cathelicidin regulates goblet cell secretions.

**Results:**

*Camp* ^*-/-*^ littermates infected with *C. rodentium* displayed increased fecal shedding and epithelial colonization. *Camp*^*-/-*^ littermates at the peak of *C. rodentium* infection (7 dpi) showed a deficient mucin layer with fewer Alcian blue/PAS filled goblet cells and a reduction in fucose (UEA-1^+^) and N-acetylglucosamine (WGA^+^) glycoproteins. By transmission electron microscopy (TEM), goblet cells in *Camp*^*-/-*^ colons were swollen and retained a large number of mucus granules during *C. rodentium* infection. *C. rodentium* infected *Camp*^*-/-*^ littermates showed impaired reactive oxygen species (ROS) production and a transcriptomic profiling associated with decreased ROS biosynthesis and an increase in ROS negative regulators. In mucin producing LS174T colonic epithelial cells, human cathelicidin LL-37 promptly induced the secretion of goblet cell-associated TFF3 and RELMβ, via a ROS-dependent mechanism.

**Conclusions:**

These findings revealed that mice lacking cathelicidin (*Camp*^*-/-*^) were more susceptible to *C. rodentium* colonization caused by defective goblet cell mucus and mucin-associated protein secretion via a ROS-dependent mechanism. Importantly, cathelicidin regulated mucus secretion revealing a non microbicidal action of this peptide with homeostatic properties on the colonic mucus barrier, critical in excluding luminal microbiota away from the epithelia to clear bacterial infections and restore gut homeostasis.

**Funding Agencies:**

NSERC

